# Antimycobacterial, Cytotoxic, and Antioxidant Activities of Abietane Diterpenoids Isolated from *Plectranthus madagascariensis*

**DOI:** 10.3390/plants10010175

**Published:** 2021-01-19

**Authors:** Kadidiatou O. Ndjoubi, Rajan Sharma, Jelili A. Badmus, Ayesha Jacobs, Audrey Jordaan, Jeanine Marnewick, Digby F. Warner, Ahmed A. Hussein

**Affiliations:** 1 Chemistry Department, Cape Peninsula University of Technology, Bellville Campus, Symphony Road, Bellville 7535, South Africa; dickakadi@yahoo.fr (K.O.N.); sharmar@cput.ac.za (R.S.); jacobsa@cput.ac.za (A.J.); 2 Applied Microbial and Health Biotechnology Institute, Cape Peninsula University of Technology, Symphony Road, Bellville 7535, South Africa; jabadmus@gmail.com (J.A.B.); marnewickj@cput.ac.za (J.M.); 3 SAMRC/NHLS/UCT Molecular Mycobacteriology Research Unit Division of Medical Microbiology, University of Cape Town, Cape Town 7700, South Africa; audrey.jordaan@uct.ac.za (A.J.); Digby.Warner@uct.ac.za (D.F.W.)

**Keywords:** *Plectranthus madagascariensis*, abietane diterpenoids, tuberculosis, antioxidant, cytotoxicity

## Abstract

Medicinal plants of the *Plectranthus* genus (Lamiaceae) are well known for their ethnomedicinal applications. *Plectranthus madagascariensis*, which is native to South Africa, is traditionally used in the treatment of respiratory conditions, scabies, and cutaneous wounds. The phytochemical studies of *P. madagascariensis* led to the isolation of five known royleanone abietanes, namely, 6β,7α-dihydroxyroyleanone (**1**), 7α-acetoxy-6β-hydroxyroyleanone (**2**), horminone (**3**), coleon U quinone (**4**), and carnosolon (**5**). The relative configuration of compound **2** was established by X-ray analysis. Compounds **1**–**4** showed antimycobacterial activity (Minimum inhibitory concentration for 90% inhibition, MIC_90_ = 5.61–179.60 μM) against *Mycobacterium tuberculosis* H_37_Rv. Compound **4** and **5** showed comparable toxicity (Concentration for 50% inhibition, IC_50_ 98.49 μM and 79.77 μM) to tamoxifen (IC_50_ 22.00 μg/mL) against HaCaT cells. Compounds **1**–**5** showed antioxidant activity through single-electron transfer (SET) and/or hydrogen-atom transfer (HAT) with compound **5** being the most active antioxidant agent. Compounds **3** and **5** were isolated for the first time from *P. madagascariensis.* The observed results suggest *P. madagascariensis* as an important ethnomedicinal plant and as a promising source of diterpenoids with potential use in the treatment of tuberculosis and psoriasis.

## 1. Introduction

Medicinal plants are fundamental components of research development in the pharmaceutical industry, whose pharmacological properties were first discovered through traditional medicine and ethnomedicinal studies [1]. From the ethnomedicinal studies done so far, natural products have been the main source in the discovery and development of many drugs. These natural products’ functional groups and diversity have exhibited their impact on drug effectiveness, optimization, safety, and the ability to prevent infectious and chronic diseases from spreading. Ethnomedicinal plants generally have smaller adverse effects, cultural acceptability, and excellent compatibility with the human body [2,3]. Regarding the impact of medicinal plants in drug discovery, enhancing the use of about 78% of new bioactive constituents can be a propitious alternative cure for infectious diseases [4]. Among the different infectious diseases present in South Africa, the airborne disease tuberculosis (TB), present in the immune system of approximately two billion people [5], is the main cause of mortality and morbidity in the country. Most of the infected people have latent TB infection, which can easily develop into active TB when associated with diabetes, psoriasis, and human immunodeficiency virus [6]. Furthermore, the risk of severe TB infection in patients affected by any of the associated diseases is twice as high as the risk for the normal population to contract either disease [7]. Additionally, the frontline TB antibiotics, namely, rifampin, streptomycin, isoniazid, ethambutol, and pyrazinamide, cause painful and disagreeable side effects such as anorexia, nausea, abdominal pain, orange/red-colored urine, skin itching, and peripheral neuropathy. Moreover, the ability of *Mycobacterium tuberculosis* (Mtb) to readily mutate has led to the spreading of multidrug resistance (MDR) to the abovementioned five frontline antituberculosis drugs. This phenomenon has limited the use of these medicines to eradicate TB, thus leading to significant health problems as the level of MDR is growing every year [6,8]. Hence, finding new or more efficient bioactive anti-TB agents with antimycobacterial and antioxidant effects is crucial. In the quest for new and effective medicines, scientists have been turning to medicines from natural resources as they are reported to have fewer side effects compared to chemically synthesized or clinical drugs [9].

**Figure 1:**
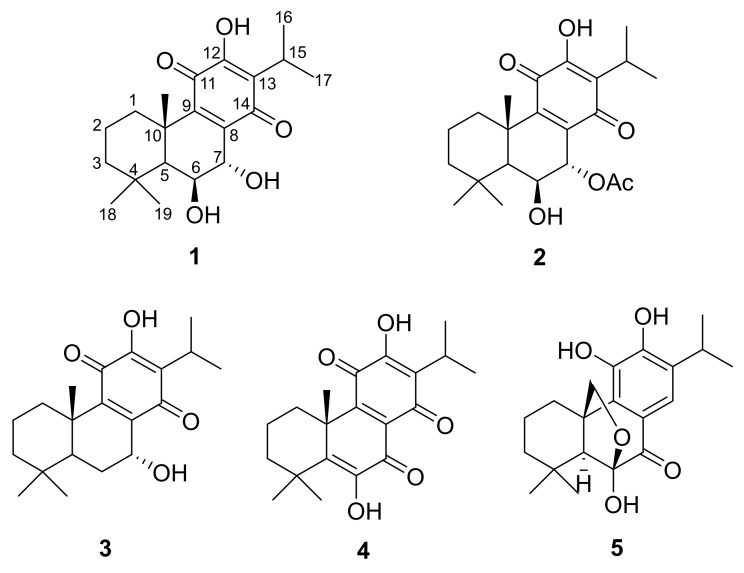
Chemical structure of compounds **1**–**5** isolated from *P. madagascariensis*.

The *Plectranthus* genus, comprising more than 300 species, belongs to the Lamiaceae family, which includes well-established medicinal plants. The ethnopharmacological applications of these plants include their use to treat infections, as well as gastrointestinal and dermatological disorders. Their medicinal properties have been attributed to the presence of bioactive diterpenes. *Plectranthus madagascariensis* (Pers.) Benth is a perennial aromatic herb native to South Africa with its traditional use in the treatment of respiratory conditions, scabies, and cutaneous wounds. Earlier chemical studies on *P. madagascariensis* showed that the plant is rich in royleanone-type abietane diterpenoids that exhibited antibacterial activities against *Bacillus subtilis, Enterococcus faecalis*, *Staphylococcus aureus*, and *Pseudomonas syringae* [10,11]. Additionally, the bioactive compounds isolated from the plant showed inhibitory effects against the enzymes α-glucosidase and butyrylcholinesterase (BuChE), as well as high selectivity for lung cancer cells NCI-H460 and NCI-H460/R [11,12]. In this study, a chemical investigation of the *P. madagascariensis* plant was done via extraction followed by the isolation and characterization of five known abietane diterpenoids (**1–5**, Figure 1). These compounds were biologically evaluated for their antimycobacterial, cytotoxic, and antioxidant activities.

## 2. Results

### 2.1. Structure Elucidation of Isolated Compounds

The phytochemical analysis of *P. madagascariensis* resulted in the isolation of five known compounds, namely, 6β,7α-dihydroxyroyleanone (**1**), 7α-acetoxy-6β-hydroxyroyleanone (**2**), horminone (**3**), coleon U quinone (**4**), and carnosolon (**5**). The NMR data of the isolated abietane diterpenoids were compared to those of previously isolated constituents from the plant and other species of the genus *Plectranthus* [13,14,15,16].

The ^1^H-NMR spectrum of compound **1** showed signals of five methyl groups at δ_H_ 1.62 (*s*, Me-20), 1.27 (*s*, Me-19), 1.06 (*s*, Me-18), 1.23 (*d*, *J* = 7.1 Hz, Me-17), and 1.23 (*d*, *J* = 7.1 Hz, Me-16). The latter two methyl groups and a proton signal at δ_H_ 3.18 (*septet*, *J* = 7.1 Hz, H-15) indicated the presence of an isopropyl group. Furthermore, the ^1^H-NMR showed signals of two low-field protons attached to two oxygenated carbons at δ_H_ 4.53 (*d*, *J* = 1.5 Hz, H-7α) and 4.46 (*brs*, H-6β), in addition to a cluster of proton signals between δ_H_ 1.20 to δ_H_ 1.50 (Table 1). The fact that the two protons at C-6β and C-7α have negligible coupling between them is directly reflected by the distortion chair form of ring B, which results from the direct effect of the planar structure of ring C and, thus, directly affects the stereochemistry of all ring B protons. The ^13^C-NMR spectrum of compound **1** showed 20 signals which were classified according to Distortionless Enhancement by Polarization Transfer (DEPT-135) as five methyl groups, three methylene groups, four methine groups, and eight quaternary carbons, with six of them forming a quinonoidal structure (ring C), at δ_C_ 140.9 (C-8), 147.6 (C-9), 183.5 (C-11), 151.2 (C-12), 124.3 (C-13), and 189.1 (C-14), in addition to the two oxygenated carbons at δ_C_ 69.3 (C-6) and 69.1 (C-7) [13].

The ^1^H-NMR spectrum of compound **2** showed signals of five upfield methyl groups at δ_H_ 1.55 (*s*, Me-20), 1.16 (*s*, Me-19), 0.92 (*s*, Me-18), 1.13 (*d*, *J* = 7.08 Hz, Me-17), and 1.11 (*d*, *J* = 7.08 Hz, Me-16), as well as the signal of a downfield methyl attached to a carbonyl group at δ_H_ 1.98 (OCOCH_3_). Two signals of two deshielded protons attached to an oxygenated carbon were observed in the ^1^H-NMR spectrum at δ_H_ 4.24 (H-6β) and 5.60 (H-7α). Moreover, a cluster of protons signals between δ_H_ 1.10 and 1.70 was observed (Table 1). Compound **2** showed a similar ^1^H-NMR profile to compound **1**, except for the low-field shift of H_7_α (from 4.53 ppm to 5.60 ppm), which indicated the link of an acetoxy group to this position. The ^13^C-NMR spectrum of compound **2** showed 22 signals, which were classified according to DEPT-135 as six methyl groups, three methylene groups, four methane groups, and nine quaternary carbons, with six of them forming a quinonoidal structure (ring C), at δ_C_ 137.0 (C-8), 150.1 (C-9), 183.3 (C-11), 151.2 (C-12), 124.3 (C-13), and 186.0 (C-14). Moreover, two oxygenated carbons at δ_C_ 66.4 (C-6), and 69.0 (C-7) and an acetoxy group at δ_C_ 21.0 (7-OCOCH_3_) and 170.1 (7-OCOCH_3_) were identified.

**Figure 2:**
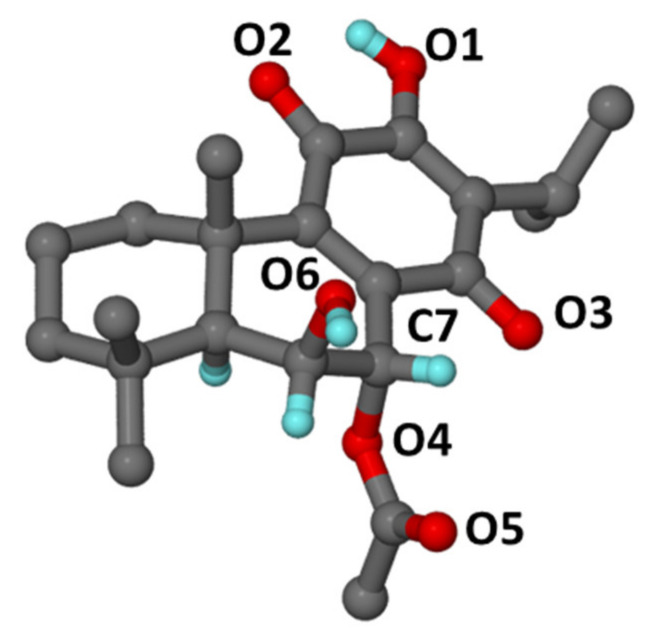
X-ray crystallographic structure of compound **2**. Selected hydrogen atoms are shown for clarity.

**Figure 3:**
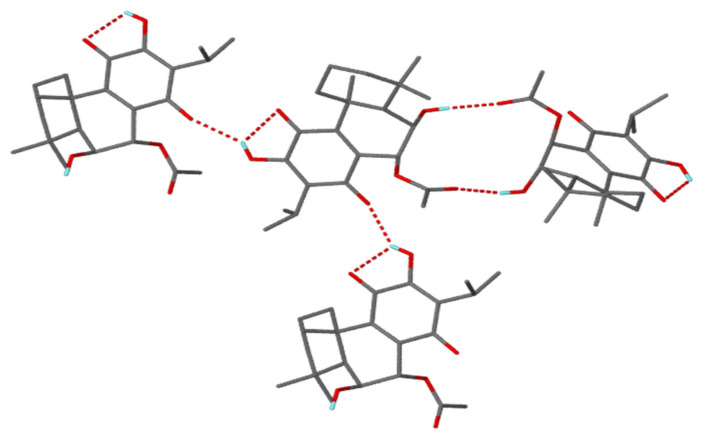
Synthon motif of compound **2**.

The stereochemistry of compounds **1** and **2** at positions 6 and 7 was reported by Kubínová et al. [11] as 6β,7β, whereas Matias et al. [12] identified them as a 6β,7α stereochemical configuration. To the best of our knowledge, no 6β,7β stereochemical configurations have been previously identified from Lamiaceae and especially *Plectranthus*; instead, 6β,7α orientations have more commonly been documented and identified. Additionally, Matias et al. [12] did not mention the isolation and/or identification of 6β,7β-related structures, but stated that compounds **1** and **2** have 6β,7α configurations without further detail. The situation becomes unclear, as Kubínová et al. [11] did not report the spectroscopic data of the two compounds in their study. The results of the X-ray analysis (Figure 2) showed that compound **2** consists of three fused cyclohexane rings with two of them as *trans*-fused cyclohexane rings which is endemic to royleanone-type abietane diterpenoids. Although the temperature for the analysis (173 K) of compound **2** was slightly higher than that used by Bernardes et al. [17] (167 K, Table 2), it was noticed that the crystalline packing showed similar $R_{2}^{2}\left(14\right)$ and $C_{1}^{1}\left(6\right)\;$ synthon motifs (Figure 3). These motifs involved the intramolecular (O1-H1A···O2) and intermolecular (O1-H1A···O3 and O6-H6A···O5) hydrogen bonds. All these parameters confirmed that compound **2** is indeed a typical 6β,7α configuration, as reported by Matias et al. [12], and is common in Lamiaceae.

The chemical structures of compounds **3**, **4**, and **5** were elucidated on the basis of a comparison of spectroscopic data to findings available in the literature.

### 2.2. Bioassay Analysis

The compounds (**1–5**) isolated from *P. madagascariensis* were tested for their antimycobacterial (Table 3), cytotoxic, and antioxidant activities (Table 4). Some of the isolated compounds, such as **1**, **2**, and **3** previously isolated from *P. grandidentatus*, showed activity against *Mycobacterium tuberculosis* H_37_Rv [18]. The 7β isomers of compounds **1** and **2**, as well as **4**, isolated from the study plant by Kubínová et al. [11], showed moderate to poor activity against a α-glucosidase inhibitor. Compounds **1–4** were found to possess cytotoxic activities against NCI-H460 (lung cancer), NCI-H460/R (lung cancer), MCR-5 (healthy lung), and MCF-7 (breast cancer) cell lines. However, compounds **1–5** were never evaluated for their cytotoxic activity against HaCaT cells, thus indicating their antipsoriatic potential. Compound **5**, previously isolated from *P. cyanus*, is an efficient antioxidant agent and is the only compound among the isolated ones which was assessed for its antioxidant activity [14].

#### 2.2.1. Antimycobacterial Assay

The antimycobacterial evaluation of compounds **1**–**5** against *Mtb* H_37_Rv showed that the media in which the cells were cultured impinged on the potency of the tested substances. As observed, the isolated compounds showed activities only in the presence of 7H9/CAS/Glu/Tx medium, except for compound **2**, which displayed moderate activities in both media (7H9/ADC/Glu/Tw and 7H9/CAS/Glu/Tx) from 7–14 days. It is to be noted that both media share the standard mycobacterial broth base, Middlebrook 7H9, and glucose (Glu) supplement. The difference lies in the use of casitone (CAS) and tyloxapol (Tx) in one medium, versus albumin–dextrose–catalase (ADC) and Tween-80 (Tw) in the other. Tx and Tw are surfactants which inhibit mycobacterial clumping. ADC, since it contains albumin (bovine albumin fraction V), is considered a useful proxy for the potential tendency of the tested compound to bind serum protein. On the other hand, CAS is a source of amino acids. The poorer activity in 7H9/ADC/Glu/Tw medium can be a result of protein binding; however, there may be other contributing factors. Moderate activity of **2** in 7H9/ADC/Glu/Tw medium may imply that the acetate group at the position 7α in ring B of its structure is liable to the inhibitory activity. It was observed that the antimycobacterial potency of the isolated compounds increased from 7–14 days, especially for compounds **3** and **4**, with (Minimum inhibitory concentration for 90% inhibition) MIC_90_ values varying from 43.19–11.93 µM and 45.41–5.61 µM, respectively.

Considering that the structure of compound **1** differs from that of compound **3** only by the presence of the β-hydroxy group at C-6, and that compound **1** (174.20 μM) had a poorer antimycobacterial activity than compound **3** (11.93 μM), it was inferred that the 6β-hydroxy group reduces the compound’s antimycobacterial potency. This observation can imply that *p*-benzoquinone ring C may have a role in the antimycobacterial activities of several quinone compounds, and that the substituents at C-6 and C-7 in ring B considerably influence the activity [18]. Compound **5** did not show activity against Mtb H_37_Rv in both media, with MIC_90_ values higher than 125 μg/mL.

#### 2.2.2. Cytotoxicity Assay

The isolated phytochemicals (compounds **1**–**5**) were tested for their cytotoxicity against HaCaT cells using the 3-(4,5-dimethylthiazol-2-yl)-2,5-diphenyltetrazolium bromide (MTT) assay to assess their antipsoriatic potential. The IC_50_ values of compounds **1**–**3** showed that compound **2** (109.38 µM) is more potent than compounds **1** (173.15 µM) and **3** (161.74 µM). These results corroborate with Matias et al. [12], who stated that the polarity and lipophilicity of the substituent at 7α position increase the cytotoxic effect of royleanone abietane diterpenoids. It was also observed that compound **3** is more active than compound **1**, which implies that the substituents at position 6 of royleanone-type abietanes can also affect the compound’s cytotoxic effect. González [19] stated that aromatic abietane diterpenoids with catechol-containing molecules and a carbonyl group at C-7, as well as coleon-type abietanes with a diosphenol moiety in ring B, displayed significant cytotoxicity activity. This statement was corroborated by compounds **4** and **5**, which were found to be the most active isolated compounds with an IC_50_ of 98.49 µM and 79.77 µM, respectively.

#### 2.2.3. Antioxidant Assay

Compound **5** is the most potent antioxidant agent, as it displayed a remarkable antioxidant inhibitory effect in all assays. The ability of compound **5** to suppress free-radical chain reactions via hydrogen-atom transfer and single-electron transfer mechanisms is attributed to the catechol groups in the benzene ring. The presence of the 12-OH group and the carbonyl group at position C-7 (*p*-position) serve as hydrogen- and/or electron-donating moieties, resulting in the formation of stable quinone derivatives. Compound **1** showed very little activity in the FRAP assay. However, in TEAC and ORAC assays, compound **1** displayed good antioxidant activities, suggesting that compound **1** neutralizes the radical cation generated by ABTS, as well as the peroxyl radicals generated from the thermal decomposition of 2,2′-azobis(2-amidinopropane) dihydrochloride (AAPH) via the hydrogen-atom transfer (HAT) mechanism [20]. Compounds **2** and **4** showed good activity in the ORAC assay and weak antioxidant activities in the TEAC and FRAP assays. None of the isolated compounds showed significant activity in the FRAP assays classified as single-electron transfer (SET) methods, except for compound **5**.

## 3. Materials and Methods

### 3.1. Sample Collection

The aerial parts of *P. madagascariensis* were collected at the Cape Peninsula University of Technology (Bellville Campus) in January 2018 and identified by Prof. Christopher Cupido of Fort Hare University, South Africa. They were registered as voucher sample (K-3, 2018) kept in the Department of Chemistry, Cape Peninsula University of Technology, Cape Town.

### 3.2. Isolation and Characterization of P. madagascariensis Phytochemicals

The fresh aerial parts of *P. madagascariensis* (2.5 kg) were cut into pieces and macerated in about 2.0 L of a solution made of hexane, dichloromethane, and acetone (2:2:1) for 1 h under frequent swirling at room temperature. After filtration, the combined extracts were evaporated to yield ~13.0 g of total extract, which was subjected to silica gel column chromatography using a hexane/ethyl acetate (Hex/EtOAc) gradient of increasing polarity. The similar fractions were combined according to their thin-layer chromatography profiles to yield 16 main fractions labeled I to XVI.

The main fraction VIII (97 mg) was purified on a Sephadex LH-20 column (5% aqueous MeOH) to give **1** (25.7 mg). Fraction VI (200 mg) was chromatographed on Silica gel using a Hex/EtOAc gradient (98:2) to yield **2** (61.9 mg). Fractions III (9.08 mg) and IV (19.82 mg) were merged (III and IV) and chromatographed on Silica gel using a Hex/EtOAc gradient (90:10) to produce **3** (13.5 mg). Fraction XI (62.21 g) was subjected to sequential chromatography on Sephadex LH-20 (5% aqueous MeOH) and then semi-preparative high performance liquid chromatography (HPLC) using acetonitrile/deionized water (60% to 80% in 30 min, then 100% acetonitrile for 15 min) to produce **4** (R_t_ 39.5 min, 6 mg). Fractions XIII and XIV (400 mg) were combined and chromatographed on Sephadex LH-20 using MeOH/deionized water (95:5) to produce **5** (26.7 mg). The structures of the isolated compounds were elucidated using NMR (Table 1). Compound **2** was analyzed using X-ray spectroscopy (Table 2).

### 3.3. Biological Assays

#### 3.3.1. Antimycobacterial Assay

The in vitro antimycobacterial activities of *P. madagascariensis* isolates against the green fluorescent protein (GFP)-tagged Mtb H_37_Rv pMSp12::GFP bioreporter strain were tested according to the standard broth microdilution method developed by Collins and Franzblau [21] and Collins et al. [22]. In this assay, the mycobacterial strain H_37_Rv was cultured in the Middlebrook 7H9 broth medium supplemented with either albumin–dextrose complex, d-glucose, and Tween-80 (7H9/ADC/Glu/Tw) or casitone, d-glucose, and tyloxapol (7H9/CAS/Glu/Tx). The minimum inhibition concentration to inhibit the growth of 90% of organisms (MIC_90_) for the tested samples was scored visually at 1 week and 2 weeks post inoculation using the microplate Green Fluorescent Protein (GFP) and expressed in µg/mL. The fluorescence was measured with excitation at 485 nm and emission at 520 nm. The media (7H9/ADC/Glu/Tw and 7H9/CAS/ Glu/Tx), as well as 5% dimethyl sulfoxide (DMSO), were used as a negative control, whereas rifampicin was used as a positive control.

#### 3.3.2. In Vitro Cytotoxicity Assay

The cytotoxicity of the isolates was tested using MTT assay reported by Mossmann [23] against the immortalized human skin epithelial keratinocytes (HaCaT) cells. The HaCaT cells were cultured in Dulbecco’s modified Eagle medium (DMEM), which was supplemented with 10% fetal bovine serum (FBS) and 1% penicillin–streptomycin solution. The HaCaT cells were grown for 4–5 days at 37 °C in a 5% carbon dioxide atmosphere. The cells were removed from the culture using the trypsin–ethylenediaminetetraacetic acid solution and transferred into a 96-well plate. The tested samples were applied to the cells and incubated at 37 °C for 24 h. Thiazolyl blue (MTT, SIGMA), dissolved in phosphate-buffered saline solution (PBS) at a final concentration of 0.8 mg/mL, was added to the cells that were exposed to the isolated compounds before incubation at 37 °C in the dark for 4 h. After observing the MTT change in color (blue to purple), the media were separated and washed with PBS. The produced formazan salts were dissolved with DMSO, and their concentrations were obtained by measuring their absorbance at 570 nm in a spectrophotometer. The IC_50_ values of the tested compounds and the positive control tamoxifen were determined using GraphPad Prism 8 software (GraphPad Software, La Jolla, CA, USA).

#### 3.3.3. FRAP (Ferric Ion Reducing Antioxidant Power) Assay

The FRAP assay was conducted according to Benzie and Strain’s [24] method, which consists of finding the compounds’ antioxidant potential in terms of μM ascorbic acid equivalents per gram dry weight (μM AAE/g). l-Ascorbic acid was used as a standard in the FRAP assay with concentrations varying between 0 and 1000 μM. The antioxidant potential of the tested samples was read at a wavelength of 593 nm in a Multiskan (Thermo Fisher Scientific, Waltham, MA, USA) spectrum plate reader.

#### 3.3.4. ORAC (Oxygen Radical Absorbance Capacity) Assay

The ORAC assay was conducted according to Prior et al.’s [25] method, which is a modified version of the original ORAC assay developed by Cao and Prior [26]. In this assay, 2,2′-azobis(2-amidinopropane) dihydrochloride (AAPH) underwent thermal decomposition to generate peroxyl radicals, resulting in a reduction in fluorescence from fluorescein. The antioxidant potential of the isolated compounds was measured by their ability to prevent any loss in fluorescence from fluorescein by neutralizing peroxyl radicals and assessing the sample’s fluorescence area under the curve (AUC). The fluoroskan spectrum plate reader was programmed to record fluorescein’s fluorescence every 2 min after the addition of AAPH with the excitation wavelength at 485 nm and the emission wavelength at 530 nm. The Trolox (TE) stock solution was used as standard with concentration varying between 0 and 417 μM. The ORAC values were calculated using a regression equation (*y* = m*x* + c) between Trolox concentration (*y* in μM) and the net area under the fluorescence decay curve (*x*). ORAC values were expressed as μM Trolox equivalents (TE) per gram of test samples (μM TE/g). The antioxidant epigallocatechin gallate (EGCG) was used as a positive control.

#### 3.3.5. TEAC (Trolox Equivalent Absorbance Capacity) Assay

The TEAC assay was done according to the method developed by Pellegrini et al. [27], which consists of determining the activities of the isolated compounds in terms of μM Trolox equivalents per gram dry weight (μM TE/g) of the test samples. Trolox was used as the standard with concentrations ranging from 0–500 μM. The absorbance was recorded by a Multiskan spectrometer plate reader at wavelength 734 nm. The antioxidant epigallocatechin gallate (EGCG) was used as a positive control.

### 3.4. Crystal Structure Analysis 

A crystal was selected for single-crystal X-ray diffraction analysis at 173 K. The diffraction data were collected on a Bruker APEX II diffractometer [28] with graphite monochromated MoKα radiation (λ = 0.71073 Å). The data were scaled and reduced with SAINT-Plus [29]. SADABS [30] was used for the absorption correction. The structure was solved by direct methods using SHELXS-97 and refined using full-matrix least-squares methods in SHELXL [31]. X-SEED [32] was used as a graphical interface. The hydrogen atoms were geometrically constrained except for those involved in hydrogen bonding, which were found in the electron density map and refined isotropically.

### 3.5. Statistical Analysis

The cytotoxic and antioxidant study experiments were done in triplicate, and data were expressed as the mean ± SD. The MIC_90_ values in antimicrobial experiments were determined using the dose–response curve (Table S1). The dose–response curve was obtained by normalizing data to the minimum and maximum inhibition controls using the Levenberg–Marquardt algorithm, from which the MIC_90_ was calculated. The IC_50_ values of the tested compounds were determined using Graph-Pad Prism 8 software (San Jose, CA, USA).

## 4. Conclusions

The phytochemical study of *P. madagascariensis* total extract resulted in the isolation of five known abietane diterpenoids (**1**–**5**). The stereochemical configuration of C6–C7 of compound **2** was finally confirmed by X-ray analysis.

The antitubercular activity of compounds **1–5** showed that the medium, in which the Mtb bacillus is cultured, affects the percentage inhibition of the compounds. Moreover, the *p*-benzoquinone ring C in quinone compounds and the substituents at C-6 and C-7 in ring B considerably influence the antimycobacterial activities. Compound **2** is the only isolated compound to show activity against Mtb H_37_Rv in 7H9/ADC/Glu/Tw medium due to the acetate group at position 7α in ring B. Compounds **4** and **3** effectively inhibit Mtb H37Rv in the 7H9/CAS/Glu/Tx medium after 14 days, with MIC_90_ values of 5.61 μM and 11.93 μM, respectively. Although not definitive, the differences in antimycobacterial activity in the two media can be instructive for medicinal chemistry teams, as well as during determinations of mechanism of action. Compounds **4** and **5** exhibited good cytotoxic activities against HaCaT cells, whereas compounds **1**–**3** showed little cytotoxicity.

The free-radical scavenging assays (ORAC, TEAC, and FRAP) done on *P. madagascariensis* phytochemicals showed that the isolated compounds suppress free-radical formation or chain reactions via HAT mechanisms of action, except for compound **5**, which operates via the SET and HAT mechanisms of action. Compounds with the ability to inhibit free radicals via both mechanisms of action showed strong antioxidant activity compared to those that operate via HAT or SET only. It was also noticed that compounds with good cytotoxicity such as compounds **2**, **4**, and **5** are good antioxidant agents, which indicates that the cytotoxic activity of the tested compounds may be mediated via an antioxidant mechanism. Moreover, compounds **2** and **4** were the only isolated abietane diterpenoids that displayed good to moderate activities in all the assays.

These findings establish *P. madagascariensis* as an important medicinal plant rich in diterpenoids which can be useful for the treatment of tuberculosis and psoriasis. It is envisaged that this study will contribute to a better understanding of the phytochemistry of *P. madagascariensis* and contribute to the development of potential antitubercular, antipsoriatic, and antioxidant compounds with improved activities.

## Figures and Tables

**Table 1:** NMR data of compounds **1**–**5** in CDCl_3_.

No. (C)	1		2		3		4		5	
	C	H, *m*, (*J* Hz)	C	H, *m*, (*J* Hz)	C	H, *m*, (*J* Hz)	C	H, *m*, (*J* Hz)	C	H, *m*, (*J* Hz)
1	38.4	2.59, *dt* (12.7, 3.0) 1.19, *m*	38.3	2.58, *dt* (12.7, 3.0) 1.22 *	35.8	2.70, *dt* (12.7, 3.0) 1.16, *m*	30.8	2.66, *m* 1.60 *	29.6	2.13, *dt* (12.7, 3.0) 2.74, *m*
2	19.0	1.61, *m* 1.83, *m*	18.9	1.59, *dt* (12.7, 3.0) 1.80, *m*	19.0	1.54, *m* 1.74, *m*	17.7	1.89, *m* 1.58 *	18.5	1.79, *m* 1.69, *m*
3	42.3	1.25, *m* 1.50, *m*	42.3	1.40, *m* 1.24 *	41.1	1.46, *m* 1.25, *m*	36.3	1.99 m 1.49 m	41.3	1.26, *m* 1.43, *m*
4	33.7		38.6		33.2		36.4		32.4	
5	49.5	1.47, *s*	49.7	1.27, *s*	45.8	1.60, *s*	143.3		58.2	1.63, *s*
6	69.3	4.46, *brs*	66.4	4.24, *s*	25.8	1.96, *d* (1.5)	146.8		105.2	
7	69.1	4.53, *d*, (1.5)	69.0	5.60, *d*, (1.8)	63.2	4.73, *d* (1.5)	177.5		192.8	
8	140.9		137.0		143.3		126.8		121.4	
9	147.6		150.1		147.8		155.1		137.7	
10	38.6		33.6		39.8		41.4		51.47	
11	183.1		183.1		183.9		183.6		140.5	
12	151.2		151.2		151.1		150.7		148.3	
13	124.3		124.3		124.2		126.0		133.3	
14	189.5		186.0		189.2		184.3		120.1	7.65, *s*
15	24.0	3.18, *septet* (7.1)	24.1	3.09, *septet* (7.1)	24.0	3.16, *septet* (7.1)	24.4	3.22, *septet* (7.0)	27.1	3.02, *septet* (7.1)
16	19.8	1.23, *d* (7.1)	19.6	1.11, *d* (7.1)	19.9	1.20, *d* (7.1)	19.8	1.24, *d* (7.0)	22.37	1.16, *d* (7.1)
17	19.9	1.23, *d* (7.1)	19.8	1.13, *d* (7.1)	19.8	1.21, *d* (7.1)	19.8	1.25, *d* (7.0)	22.5	1.17, *d* (7.1)
18	33.5	1.06, *s*	33.5	0.92, *s*	33.1	0.98, *s*	27.2	1.43, *d* (3.9)	33.7	1.04, *s*
19	24.3	1.27, *s*	23.6	1.16, *s*	21.7	0.90, *s*	29.1	1.42, *d* (3.9)	22.2	1.31, *s*
20	21.6	1.62, *s*	21.3	1.55, *s*	18.4	1.22, *s*	27.5	1.64, *s*	72.0	3.37, *d* Hα, (7.5) 4.29, *d* Hβ (7.5)
7-OCOC$H_{3}$			21.0	1.98, *s*						
7-OCOC$H_{3}$			170.1							
6-OH		5.31, *s*						7.09, *s*		5.21, *s*
7-OH						3.03, br				
12-OH				7.23, *s*		7.26, *s*		7.08, *s*		7.23, *s*

* Overlapping peaks in the same column.

**Table 2:** Single-crystal X-ray parameters of 7α-acetoxy-6β-hydroxyroyleanone (**2**).

Parameters	Values	
Identification code	**2**	Bernardes et al. [17]
Molecular formula	C_20_H_30_O_6_	C_20_H_30_O_6_
Temperature	173 K	167 K
Crystal size	0.290 × 0.360 × 0.400 mm	0.25 × 0.20 × 0.500 mm
Crystal system	Orthorhombic	Orthorhombic
Space group	P2_1_2_1_2	P2_1_2_1_2
Unit cell dimensions		
a	14.115(3) Å	14.0964 (12) Å
b	20.620(4) Å	20.5705 (18) Å
c	7.3893(15) Å	7.3873(7) Å
Volume	2150.7(8) Å^3^	2142.1 (3) Å^3^
Reflections collected	32427	10019
Final R (*I* > 2σ(*I*))	R_1_ = 0.0385; wR_2_ = 0.0937	R_1_ = 0.0485; wR_2_ = 0.0843
R indices (all data)	R_1_ = 0.0451; wR_2_ = 0.0979	R_1_ = 0.0840; wR_2_ = 0.0961

**Table 3:** The antimycobacterial activities of the isolated compounds at 90% inhibition in 7H9/ADC/Glu/Tw and 7H9/CAS/ Glu/Tx media.

Identification Code ^1^	7H9/CAS/ Glu/Tx (μg/mL)		7H9/ADC/Glu/Tw (μg/mL)	
	7 Days	14 Days	7 Days	14 Days
III and IV	>125	>125	>125	>125
VI	31.25	31.864	63.095	>125
VIII	16.189	14.71	>125	>125
XI	16.043	7.318	>125	>125
XIII and XIV	64.241	14.375	>125	>125
Total extract	31.42	16.04	>125	>125
**1**	62.5 (179.60 µM)	60.62 (174.20 µM)	>125	>125
**2**	15.22 (39.02 µM)	15.63 (40.08 µM)	14.36 (36.82 µM)	14.64 (37.54 µM)
**3**	14.34 (43.19 µM)	3.96 (11.93 µM)	>125	>125
**4**	15.62 (45.41 µM)	1.93 (5.61 µM)	>125	>125
**5**	>125	>125	>125	>125
Positive Control (Rifampicin)	0.015 (0.02 µM)	0.035 (0.04 µM)	0.001 (0.001 µM)	0.002 (0.002 µM)

^1^ Under identification code, the roman numerals represent the respective chromatographic fractions of the total extract, whereas the numbers represent the isolated compounds.

**Table 4:** The cytotoxic and antioxidant activities of the *P. madagascariensis* isolated compounds.

	Cytotoxicity Activity (HaCaT)	Antioxidant Activities		
Identification Code of Samples	IC_50_ (µg/mL) ^1^	TEAC (µM TE/g) ^2^	FRAP (µM AAE/g) ^3^	ORAC (µM TE/g) ^4^
Total extract	28.18 ± 2.42	N/A	N/A	N/A
**1**	60.25 ± 3.95 (173.15 µM)	5080.8 ± 0.04	541.5 ± 1.59	23,625.0 ± 1.64
**2**	42.66 ± 1.22 (109.38 µM)	3700.8 ± 1.50	345.5 ± 0.77	21,404.1 ± 4.35
**3**	53.70 ± 0.67 (161.74 µM)	Inactive	296.5 ± 6.72	12,443.3 ± 3.61
**4**	33.88 ± 1.43 (98.49 µM)	225.5 ± 1.47	2109.6 ± 2.78	21,857.8 ± 5.85
**5**	27.52 ± 2.32 (79.77 µM)	4876.3 ± 0.49	13,772.2 ± 2.76	29,287.4 ± 4.75
Positive control ^5^	22.00 (59.30 µM)	4722.5 ± 2.22	10,455.1 ± 0.81	14,970.0 ± 5.53

^1^ IC_50_ (µg/mL), half maximal inhibitory concentration. ^2^ TEAC (µM TE/g), Trolox Equivalent Absorbance Capacity expressed in terms of μM Trolox equivalents per gram. ^3^ FRAP (µM AAE/g), Ferric Ion Reducing Antioxidant Power expressed in terms of μM ascorbic acid equivalents per gram. ^4^ ORAC (µM TE/g), Oxygen Radical Absorbance Capacity expressed as μM Trolox equivalents (TE) per gram. ^5^ The positive control for cytotoxicity was tamoxifen, and that for antioxidant activities was epigallocatechin gallate (EGCG).

## Data Availability

CCDC 2054963 contains the supplementary crystallographic data for 7α-acetoxy-6β-hydroxyroyleanone (compound 2). The data can be obtained free of charge from The Cambridge Crystallographic Data Centre via www.ccdc.cam.ac.uk/data_request/cif.

## Supplementary Materials

The following are available online at https://www.mdpi.com/2223-7747/10/1/175/s1. Table S1: Antimycobacterial response curves.
